# A new Brief computerized cognitive screening battery (CompCogs) for early diagnosis of Alzheimer’s disease

**DOI:** 10.1590/S1980-57642009DN20100004

**Published:** 2008

**Authors:** Helenice Charchat Fichman, Ricardo Nitrini, Paulo Caramelli, Koichi Sameshima

**Affiliations:** 1 Department of Psychology, Catholic University, Rio de Janeiro, Brazil.; 2 Department of Neurology, University of São Paulo School of Medicine, São Paulo, Brazil.; 3 Department of Internal Medicine, Federal University of Minas Gerais, Belo Horizonte, Minas Gerais, Brazil.; 4 Department of Neurology and Discipline of Medical Informatics, Department of Pathology, University of São Paulo School of Medicine, São Paulo, Brazil.

**Keywords:** Alzheimer’s disease, dementia, computerized neuropsychological tests, brief cognitive battery, doença de Alzheimer, demência, testes neuropsicológicos computadorizados, bateria cognitiva breve

## Abstract

Screening tests for early diagnosis of dementia are of great clinical relevance. The ideal test set must be brief and reliable, and should probe cognitive components impaired in Alzheimer’s disease (AD).

**Objectives:**

To develop a new Computerized Cognitive Screening test (CompCogs), and to investigate its validity for the early diagnosis of AD, and evaluate its heuristic value in understanding the processing of information in AD.

**Methods:**

The computerized neuropsychological performance battery, originally including six tests, was applied in forty seven patients with probable mild AD and 97 controls matched for age and education. This computerized neuropsychological test battery, developed with MEL Professional, allows control of timing and order of stimuli presentation, as well as recording of response type and latency. A brief-screening version, CompCogs, was selected using the most discriminative neuropsychological test variables derived from logistic regression analysis. Full battery administration lasted about 40 minutes, while the CompCogs took only 15 minutes.

**Results:**

CompCogs included the Face test (correct response) and Word and Forms with Short term memory tests (reaction time). CompCogs presented 91.8% sensitivity and 93.6% specificity for the diagnosis of AD using ROC analyses of AD diagnosis probability derived by logistic regression.

**Conclusions:**

CompCogs showed high validity for AD early diagnosis and, therefore, may be a useful alternative screening instrument.

## Abstract (pt)

Testes de rastreio para o diagnóstico precoce de demência são de grande relevância clínica. O teste ideal deve ser breve e confiável e inclui componentes cognitivos comprometidos na Doença de Alzheimer (DA).

**Objetivo:**

Desenvolver um novo teste computadorizado de rastreio cognitivo (CompCogs), investigar a sua validade para o diagnóstico inicial DA e avaliar o seu valor heurístico na compreensão da velocidade de processamento na DA.

**Métodos:**

O desempenho na bateria de testes neuropsicológicos computadorizados, originalmente composta de seis testes, foi avaliado em quarenta e sete pacientes com diagnóstico de DA clinicamente provável em estágio inicial e 97 controles pareados por idade e anos de escolaridade foram estudados. A bateria de testes neuropsicológicos computadorizados, desenvolvida com o programa *MEL professional*, permite o controle do tempo e ordem de apresentação dos estímulos, bem como, tipo e latência de resposta. A versão breve para rastreio cognitivo, CompCogs, foi selecionada utilizando as variáveis neuropsicológicas com maior poder discrimintativo derivadas da análise de regressão logística. Toda a bateria tem duração de aproximadamente 40 minutos e o CompCogs apenas 15 minutos.

**Resultados:**

CompCogs incluiu o teste Face (resposta correta), os testes Palavras e Formas com memória de curto prazo (tempo de reação). O CompCogs apresentou 91,8% de sensibilidade e 93,6% de especificidade baseado na análise da curva ROC da probabilidade do diagnóstico de DA derivada da análise de regressão logística.

**Conclusões:**

CompCogs mostrou alta validade para o diagnóstico da DA, portanto é um instrumento alternativo útil para rastreio cognitivo.

Demographic studies have described progressive and significant increase in the elderly population over recent years.^1^ Advances in medical knowledge together with the implementation of adequate health care related infrastructure for the population are raising life expectancy of the world population. One of the forecast scenarios is an important increase of dementia prevalence with consequent need for health care expenditure.

Dementia is a syndrome characterized by decline of memory function associated with other neuropsychological changes, with increased incidence on aging.^2,3^ There are about 70 diseases associated with dementia and in this wide range of etiologies, Alzheimer’s disease (AD) is the most frequent cause.^3,4^ For these reasons, AD is expected to become an increasingly important public health problem in the coming decades, and therefore, its early diagnosis may prove crucial for adequate disease management and to introduce preventive measures or to retard its progression.

Neuropsychological testing is fundamental for early clinical diagnosis of AD. The currently available tools for cognitive screening in AD are based on pencil-and-paper tests. These tests evaluate memory function alone or are combined with other neuropsychological function investigation, such as attention, verbal fluency, naming, working memory, visuo-spatial abilities, temporal/spatial orientation, and language.^4-15^ Recently, several investigators have developed computerised neuropsychological tests for dementia diagnosis to study subtle cognitive impairment in elderly population, and also to evaluate therapeutic drug efficacy.^16-25^ These computerized test batteries, which usually assess memory, reaction time, and other cognitive functions, tend to be very lengthy.^16-25^

The most evident advantages of computer-based neuropsychological examination are the precise time control on stimulus presentation, and the accurate measurement of motor response latency^27-28^. In this study we exploited a software technology that allows millisecond accuracy and resolution for the presentation of visual stimuli as well as for motor response latency measurement. This level of accuracy and resolution is practically unattainable using paper-and-pencil tests, even with the aid of a chronometer.^27,28^

Another common limiting characteristic of the majority of non-computerized tests is the availability of only a single version of the application form. The repeated application of one test set to the same patients becomes unsuitable for monitoring clinical evolution of cognitive functions, because testing performed within short time intervals can be affected by the learning effect.^26-28^ The computerized tests, however, can store and generate a large number of stimulus sets, and, for each test, it can randomly select a subset of these stimuli. In the investigation of degenerative diseases, such as AD, repeated evaluations within short time intervals are essential for the prospective confirmation of the diagnosis or for the assessment of disease progression.

Three main limitations of computerized neuropsychological tests are:

1) difficulty in evaluating and analysing oral answers;
2) necessity to examine subject interaction with the computer to detect any problems in understanding instructions; and
3) lack of exhaustive validation, because most tests are only used in research protocols.^26-29^

In this study we developed the Computerized Cognitive Screening test (CompCogs) and investigated its validity as an alternative supporting instrument for the early detection of AD. The CompCogs, compared to the traditionally used neuropsychological tests, has the natural advantages and limitations resulting from the use of a computer in the whole testing procedure. Since CompCogs is briefer than other computerized neuropsychological batteries, it could prove to be a useful dementia screening test.

## Methods

### Subjects

All subjects gave written informed consent according to the research protocol (approved by the Ethics Committee of the Hospital das Clínicas of the University of São Paulo School of Medicine). Forty seven patients with the diagnosis of probable AD, as defined by the National Institute of Neurological and Communicative Disorders and Stroke – Alzheimer’s Disease and Related Disorders Association (NINCDS-ADRDA) criteria,^30^ participated in this study. All patients had mild dementia (CDR 1) as defined by the Clinical Dementia Rating (CDR) Scale.^31^ For diagnostic characterization, patients were submitted to neurological examinations, laboratory exams, and to a non-computerised neuropsychological assessment that included:

1) Mattis Dementia Rating Scale (DRS);
2) animal and FAS verbal fluency;
3) Clock drawing;
4) copy and 30 minutes recall of Rey Complex Figure;
5) Rey Auditory Verbal Learning Test (RAVLT), and
6) digit span.

These patients were compared to a group of 97 elderly subjects with no current or past history of neurological or psychiatric diseases, without complaints of memory loss, and fully independent in the performance of daily living activities. The control group was only submitted to the DRS to rule out cognitive impairment. The two groups were matched by age and years of education (Table 1). All subjects were right-handed and literate. Subjects using drugs acting on the central nervous system were not included in the control group.

**Table 1:** Age and years of education in patients with Alzheimer's disease and control subjects.

	Controls (N=97) Mean (SD)	AD patients (N=47) Mean (SD)	p*
Age (years)	69.46 (6.19)	72.03 (5.60)	p>0.05
Education (years)	6.30 (4.91)	9.15 (5.15)	p>0.05
MMSE	29.07 (1.66)	20.32(2.62)	p>0.05

* Student "t" test.

### Material

We implemented the computerized cognitive test battery (Brazilian Portuguese version),^20,21^ composed of six neuropsychological choice reaction time tests, using MEL Professional version 2.0 software (Psychological Software Tools).^32^ This was run on an IBM-PC compatible microcomputer using a 14-inch SVGA colour monitor for visual stimulus presentation. The sequence of stimulus presentation and the recording of response and reaction time (RT) measurements were entirely controlled by the computer system. Response latencies were measured with one millisecond resolution. A keypad with five buttons, labelled from 1 to 5, was used as a response input device. The neuropsychological tests were applied in a light-controlled room with acoustic attenuation.

### Procedures

All subjects were submitted to the computerised neuropsychological test battery. The application of all six tests took approximately 40 minutes. The general procedure for each of the six tests was as follows (for more detail see Charchat, 1999, 2001):^20,21^

#### Face test

1) Oral and written instructions: “ Unknown faces will be presented. Watch carefully!”. 2) Ten drawings of unknown faces were presented on the screen for 10 seconds. 3) Oral and written instructions: “Now, faces will be presented again. Some you have seen in the first screen and others are new. If a face you have seen before appears, press button 1, otherwise press 3. Press the button as quickly as you can.” 4) Twenty faces (10 previously presented and 10 distracters) are sequentially presented at centre screen in random order. If the subject does not press a button for 10 seconds, the next face is presented. 5) When button 1 is pressed for the faces previously shown in the first screen, or button 3 for distracters, the answer is considered correct. For this and all other tests, response latencies were also recorded.

#### Picture test

This test follows the same procedure described for the Face Test, using pictures instead of faces as a stimulus set. All steps were repeated three times in assessing the learning effect, using the same set of pictures.

#### Word test

The Word test was similar to the Face and Picture tests, using a word list as a stimulus set. Following the procedure adopted for the Picture test, this test was repeated three times to assess the learning effect.

#### Direct form test

1) Oral and written instructions: “A pair of geometric forms will be presented. If they are the same, press button 1, otherwise press 3. Press the buttons as quickly as you can.” 2) A pair of geometric forms (square, circle or triangle) is simultaneously presented side-by-side at the centre of computer screen. If the subject does not press a button for 10 seconds, the next geometric form pair is presented. Step 2 is repeated 50 times. 3) If the subject pressed button 1 for the same geometric form pair or button 3 for the different pair, the answer was considered correct. These response latencies were registered.

#### Forms with short-term memory (STM) test

**Figure 1:**
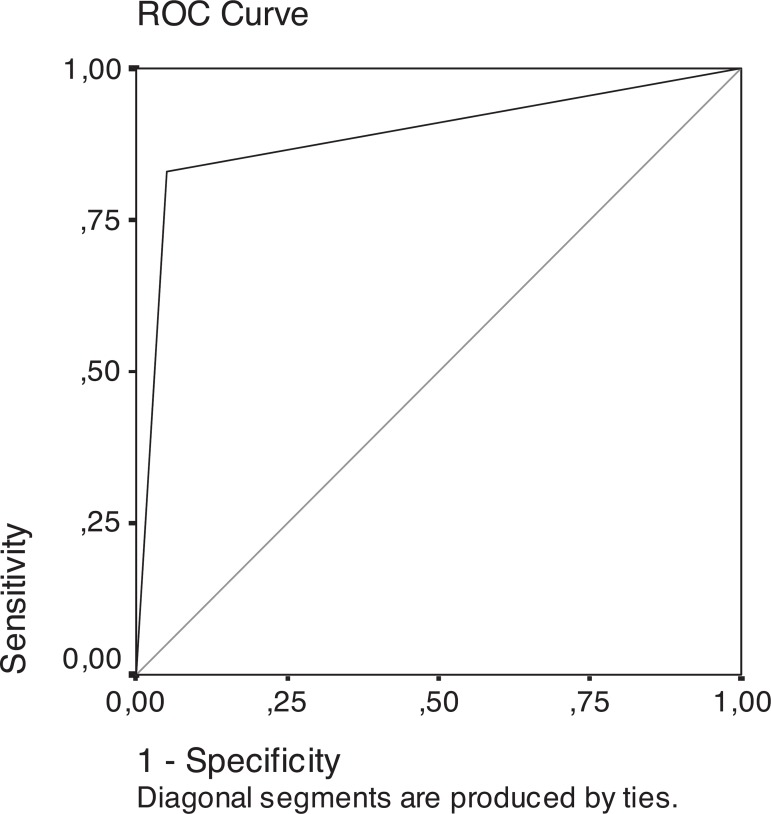
ROC curve generated by the logistic regression probability of subjects being classified as AD.

1) Oral and written instruction: “Watch carefully! A geometric form will be presented on the screen, then the image will be erased and another geometric form will appear. If they are the same, press button 1, otherwise press button 3. Press the buttons as quickly as possible.” 2) A randomly chosen geometric form (a square, circle or triangle) is presented at the centre of the computer screen for one second, then after a one second delay another geometric form is presented. 3) If the subject pressed button 1 for the same geometric form pair or button 3 for the different pair, the answer was considered correct. 4) If the subject did not press a button for 10 seconds, the next geometric form pair was presented (see Figure 1 for further details). 5) Steps 2, 3 and 4 were repeated 50 times. The motor response latencies were also measured and stored.

#### Number test

1) Oral and written instructions: “A number will be presented on the computer screen, press the button corresponding to this number on the response box as quickly as possible.” 2) At the beginning of each trial, a warning tone is presented, then after 300 ms a randomly selected number (1, 2, 3, 4 or 5 with equal probability) is displayed at the centre of the computer screen. 3) The number is displayed until a button is pressed. If the subject does not press a button for 10 seconds, the next trial is started. 4) Steps 2 and 3 were repeated 100 times. 5) If the subject had pressed the button corresponding to the number on the screen, the answer was considered correct. The motor response latencies were also recorded.

The following testing sequence was applied:

1) Direct Form Test;
2) Forms with STM Test;
3) Face Test;
4) Word Test;
5) Number Test;
6) Picture Test.

### Data analysis

For analyses, the total number of correct responses was converted to percentage of correct response (PCR), and the average response latency measured in milliseconds was log-transformed. A ROC (Receiver Operating Characteristic) analysis for each variable was conducted. The stepwise forward algorithm with likelihood-ratio criteria was used to generate the logistic regression model including the cognitive variables. This regression produced a new variable with the probabilities that each case has AD diagnosis. ROC analysis of this probability variable was conducted to find the best cut off to classify the cases using CompCogs.

## Results

The ROC curve for each computerized neuropsychological test variable showed area under the curve higher than 0.800 in all variables of reaction time (RT) and percentage of correct response (PCR) in episodic and short-term memories. The ROC curve analysis is presented in Table 2.

**Table 2:** ROC curve for different cognitive variables.

Cognitive variables	AUC
PCR Word Test	0.964
RT Word Test	0.932
PCR Face Test	0.898
RT Number Test	0.867
RT Forms with STM Test	0.866
PCR Picture Test	0.852
RT Picture Test	0.808
PCR Forms with STM Test	0.807
RT Face Test	0.806
RT Direct Forms Test	0.800
PCR Direct Forms Test	0.673
PCR Numbers Test	0.583

AUC: area under curve; PCR: percentage of correct response; RT: reaction time.

The logistic regression was generated showing that the percentages of correct responses on the Face Test, Reaction time log transformed on Word and Form with STM tests produced the best model with better adjustment, and yielded the highest percentage of correct diagnostic classification. This model showed 14.93 of –2 log likelihood adjustment. The best-adjusted logistic model function was:

(1) $$P\left(\mathrm{AD}\right)=\frac{1}{1+\exp\left(-43.91-0.09x+8.51y+6.34z\right)}$$

where *x* is the percentage of correct identification of face (PCR Faces), *y* reaction time log transformed identification of Words, and *z* reaction time log transformed Form with STM. This expression indicates the probability of an individual being an AD patient, based on these cognitive variables. The logistic analysis showed that the adjusted coefficients of the variables were significant. The probability variable ROC analysis showed 91.8% sensitivity and 93.6% specificity with the cut-off 0.29. Figure 1 shows the ROC curve of probability.

## Discussion

The logistic regression method selected the three most discriminative variables (percentage of correct responses on Face, reaction time of Word and Form with STM tests) to compose the computerized brief-screening version called CompCogs, which attained high sensitivity (93.6%) and specificity (91.8%) as a screening test for early AD diagnosis. The CompCogs is brief and focuses on the main cognitive components impaired in AD (episodic memory and speed of information processing), confirming the findings of previous studies using all neuropsychological computerized tests, that the AD group presented increased reaction time in all choice RT tests compared with the control group.^20,21,25^

These results suggest that AD promotes a slowing in information processing, being in agreement with other studies^32,33^ which, using different procedures, also observed increased RT in the early stages of AD. Moreover, the significant increase of RT in AD patients on all tasks reinforces the hypothesis of the functional linearity and generality of RT.^32^ According to this hypothesis, AD generates a slowing of cognitive processing which is essentially independent of the nature of the task or the cognitive functions involved.

In this context, it was possible to hypothesize that RT underlies all cognitive functions, because its measure assesses, at least in part, the speed at which the information is processed by the central nervous system, independently of the accuracy of this processing. Regarding the number of correct responses in choice reaction time tests, the AD group showed significant reduction only in the Forms with STM test and in the episodic memory test. This result suggests that, despite the slowing in information processing, the patients did not present visual perceptual deficits. The impairment was only observed in the tasks that demanded, besides visual perception, memory abilities.

In summary, the computerized neuropsychological test battery does not include solely the evaluation of episodic memory or a test of global screening. By including the speed of information processing, it highlights the importance of a neuropsychological marker not explored by the majority of diagnostic tools currently available.^5-13^

The nature of the tasks, socio-demographic characteristics of the samples, statistical methods and absence, in majority of studies, of test validation on a different sample constitute limitations that prevent adequate comparison of the sensitivity and specificity of CompCogs values with other studies. Despite these limitations, the CompCogs sensitivity and specificity values were similar, or superior, to recent results of studies using neuropsychological test batteries for the diagnosis or screening of dementia.^16,17,23,24^

The strictness in the selection of the groups and the attempt to not include patients with doubtful diagnosis made the model less sensitive to the individuals who are lie on the borderline of normal and pathological aging processes. Specific episodic memory deficits can also occur in other conditions, such as mild cognitive impairment (MCI), depression or pre-clinical stages of AD, and in persons with low educational level or advanced age.^8,10,11,14,15,34^

The diagnostic value of CompCogs in detecting very mild AD or MCI was not investigated in the present study. Similarly, CompCogs was used for screening AD whereas other types of dementia were not investigated. Cultural and educational aspects were also not investigated in this study. Since our long-term goal was to develop a brief computerised test that could be used specifically for screening the general population to detect mild AD, further investigations are necessary to evaluate and improve the screening test. Future studies involving larger population groups including patients with MCI and other types of dementia which also explore different cultural and educational samples should be conducted.

In conclusion, the CompCogs, as a brief cognitive instrument, showed high sensitivity and specificity for the diagnosis of early AD and could be a useful screening tool in the clinical practice. This screening task had the advantage of measuring speed of information processing using reaction time, a variable not investigated by majority of other cognitive batteries.

## References

[1] United Nations (1986). Report of the Interregional Seminar to promote the implementation of the
International Plan of Action on aging.

[2] Amaducci LA, Lippi A, Maurer K, Riederer P, Beckmann H (1990). Descriptive and analytic epidemiology of Alzheimer's
disease. Alzheimer's Disease. Epidemiology, Neuropathology, Neurochemistry, and
Clinics.

[3] Nitrini R, Mathias SC, Caramelli P (1995). Evaluation of 100 patients with dementia in Sao Paulo, Brazil:
correlation with socioeconomic status and education. Alzheimer Dis Assoc Disord.

[4] Fratiglioni L, Grut M, Forsell Y (1991). Prevalence of Alzheimer disease and other dementias in an elderly
urban population: Relationship with age, sex, and education. Neurology.

[5] Nitrini R, Lefévre BH, Mathias SC (1994). Brief and easy to administer neuropsychological tests in the
diagnosis of dementia. Arq Neuropsiquiatr.

[6] Porto SC, Charchat-Fichman H, Caramelli P, Bahia VA, Nitrini R (2003). Dementia Rating Scale - DRS - in the diagnosis of patients with
Alzheimer´s dementia. Arq Neuropsiquiatr.

[7] Lorentz WJ, Scanlan JM, Borson S (2002). Brief screening tests for dementia. Can J Psychiatry.

[8] Nitrini R, Caramelli P, Herrera E Jr (2004). Performance of illiterate and literate nondemented elderly
subjects in two tests of long-term memory. J Int Neuropsychol Soc.

[9] Takada LT, Caramelli P, Fichman HC (2006). Comparison between two tests of delayed recall for the diagnosis
of dementia. Arq Neuropsiquiatr.

[10] Nitrini R, Caramelli P, Porto CS (2007). Brief cognitive battery in the diagnosis of mild Alzheimer´s
disease in subjects with medium and high levels of education. Dem Neuropsychol.

[11] Mitrushina M, Uchiyama C, Satz P (1995). Heterogeneity of cognitive profiles in normal aging: implication
for early manifestation of Alzheimer's disease. J Clin Exp Neuropsychol.

[12] Solomon PR, Hirschoff A, Kelly B (1998). A 7 minute neurocognitive screening battery highly sensitive to
Alzheimer's disease. Arch Neurol.

[13] Buschke H, Kuslansky G, Katz M (1999). Screening for dementia with the memory impairment
screen. Neurology.

[14] Ritchie K, Leibovici D, Ledésert B, Touchon J (1996). A typology of sub-clinical senescent cognitive
disorder. Br J Psychiatry.

[15] Ritchie K, Ledésert B, Touchon J (2000). Subclinical cognitive impairment: Epidemiology and clinical
characteristics. Compr Psychiat.

[16] Robbins TW, James M, Owen AM, Sahakian BJ, McInnes L, Rabbitt P (1994). Cambridge Neuropsychological Test Automated Battery (CANTAB): a
factor analytic study of a large sample of normal elderly
volunteers. Dementia.

[17] Robbins TW, James M, Owen AM (1998). A study of performance on tests from the CANTAB battery sensitive
to frontal lobe dysfunction in a large sample of normal volunteers:
implications for theories of executive functioning and cognitive
aging. J Int Neuropsychol Soc.

[18] Veroff AE, Cutler N, Sramek J, Prior P, Mickelson W, Hartman J (1991). A new assessment tool for neuropsychopharmacologic research: the
Computerized Neuropsychological Test Battery. J Geriatr Psychiatry Neurol.

[19] Veroff AE, Bodick NC, Offen WW, Sramek JJ, Cutler NR (1998). Efficacy of xanomeline in Alzheimer disease: Cognitive
improvement measured using the Computerized Neuropsychological Test Battery
(CNTB). Alzheimer Dis Assoc Dis.

[20] Charchat H, Nitrini R, Caramelli P, Sameshima K (2001). Investigação de marcadores clínicos dos
estágios iniciais da doença de Alzheimer com testes
neuropsicológicos computadorizados. Rev Psicol: Refl Crít.

[21] Charchat H Desenvolvimento de uma bateria de testes neuropsicológicos
computadorizados para o diagnóstico precoce da Doença de
Alzheimer.

[22] Karczyn AD, Aharonson V (2007). Computerized methods in the assessment and prediction of
dementia. Curr Alzheimer Res.

[23] Doniger GM, Dowolatsky T, Zucher DM, Chertkow H, Schweiger A, Simon ES (2006). Computerized cognitive testing battery identifies mild cognitive
impairment and mild dementia even in the presence of depressive
symptoms. Am J Alzheimers Dis Other Dem.

[24] Doniger GM, Zucker DM, Schweiger A (2005). Towards practical cognitive assessment for detection of early
dementia: a 30 minute computerized battery discriminates as well as longer
testing. Curr Alzheimer Res.

[25] Caramelli P, Chaves ML, Engelhard E (2004). Effects of galantamine on attention and memory in Alzheimer´s
disease measured by computerized neuropsychological tests: results of the
Brazilian Multi-Center Galantamine study (GAL-BRA-01). Arq Neurpsiquiatr.

[26] Meulen EF, Schmand B, van Campen JP (2004). The seven minute screen: a neurocognitive screening test highly
sensitive to various types of dementia. J Neurol Neurosurg Psychiatry.

[27] Kay GG, Starbuck VN, Maruish ME, Moses Jr JM (1997). Computerized neuropsychological assessment. Clinical Neuropsychology: Theoretical Foundations for
Practitioners.

[28] Mead AD, Drasgow F (1993). Equivalence of computerized and paper-and-pencil cognitive
ability tests: a meta-analysis. Psych Bull.

[29] Butcher JN, Perry JN, Atlis MM (2000). Validity and utility of computer-based test
interpretation. Psychol Assess.

[30] McKhann G, Drachman D, Folstein M, Katzman R, Price D, Stadlan EM (1984). Clinical diagnosis of Alzheimer's disease: report of the
NINCDS-ADRDA work group under the auspices of the Department of Health and
Human Services Task Force on Alzheimer's disease. Neurology.

[31] Hudges CP, Berg L, Danzinger WL, Coben LA, Martin RL (1982). A new clinical scale for the staging of dementia. Br J Psychiatry.

[32] Schneider W (1995). MEL Professional User's Guide.

[33] Mitrushina M, Satz P, Drebing C (1994). The differential pattern of memory deficit in normal aging and
dementias of different etiology. J Clin Psychol.

[34] Kerr B, Calogero M, Vitiello MV, Prinz PN, Williams DE, Wilkie F (1992). Letter matching: Effects of age, Alzheimer's disease, and major
depression. J Clin Exp Neuropsychol.

[35] Nebes RD, Brady CB (1992). Generalized cognitive slowing and severity of dementia in
Alzheimer's disease: implications for the interpretation of response-time
data. J Clin Exp Neuropsychol.

[36] Hofman M, Seifritz E, Kräuchi K (2000). Alzheimer's disease, depression and normal ageing: Merit of
simple psychomotor and visuospatial tasks. Int J Geriatr Psychiatry.

[37] Baddeley AD, Hitch GJ, Bower G (1974). Working memory. Recent Advances in Learning and Motivation.

[38] Morris RG, Baddeley AD (1988). Primary and working memory functioning in Alzheimer-type
dementia. J Clin Exp Neuropsychol.

[39] Baddeley AD, Logie R, Bressi S, Della Sala S, Spinnler H (1986). Dementia and working memory. Q J Exp Psychol Sect A-Hum Exp Psychol.

[40] Baddeley AD, Bressi S, Della Sala S, Logie R, Spinnler H (1991). The decline of working memory in Alzheimer's disease: A
longitudinal study. Brain.

[41] Caramelli P, Poissant A, Gauthier S (1997). Educational level and neuropsychological heterogeneity in
dementia of the Alzheimer type. Alzheimer Dis Assoc Dis.

